# BEST PRACTICES AND DATA EXEMPLARS IN DESIGN AND EVALUATION OF EARLY-PHASE DYADIC INTERVENTIONS

**DOI:** 10.1093/geroni/igae098.1863

**Published:** 2024-12-31

**Authors:** Karen Lyons, Joan Monin, Steven Zarit

**Affiliations:** Chair; Co-Chair; Discussant

## Abstract

Despite growing numbers of early-phase dyadic interventions, few move beyond early-phase and often lack intentional plans for development and evaluation to optimize success. This session includes four data-based papers that will discuss essential elements of design and evaluation of early-phase dyadic interventions with older adults and their care partners in both cognitive impairment and chronic illness contexts. First, Dr. Joan Monin and colleagues will describe the advantages of, and phases involved in, using an Appreciative Inquiry Workshop approach to develop an attachment-based communication intervention with dyads living with dementia. Second, Dr. Kalisha Bonds Johnson and colleagues will present phase I data from a study to develop and test a formal care decision-making intervention for African-American parent-adult child dyads living with dementia. The paper will discuss the importance of intentional community engagement, use of theory, and type of dyad (e.g., mother-daughter) when designing dyadic interventions. Third, Dr. Michael McCarthy and colleagues will use data from several studies to exemplify the advantages of mixed-methods designs in developing dyadic interventions, particularly in rural samples (e.g., strengthening participant engagement, maximizing resources, surfacing new questions). Finally, Dr. Karen Lyons and colleagues will present two approaches to evaluating dyadic benefits of interventions in small samples, the importance of assessing pre-test risk status within care dyads, and the need to assess relational impact of behavioral interventions. Speakers will share best-practices in developing, designing and evaluating early-phase dyadic interventions followed by a discussion of key take-homes by Dr. Steve Zarit.

